# MoS_2_-Based Photodetectors Powered by Asymmetric Contact Structure with Large Work Function Difference

**DOI:** 10.1007/s40820-019-0262-4

**Published:** 2019-04-16

**Authors:** Zhe Kang, Yongfa Cheng, Zhi Zheng, Feng Cheng, Ziyu Chen, Luying Li, Xinyu Tan, Lun Xiong, Tianyou Zhai, Yihua Gao

**Affiliations:** 1 Center for Nanoscale Characterization and Devices (CNCD) Wuhan National Laboratory for Optoelectronics (WNLO) and School of Physics and School of Materials Science and Engineering, Huazhong University of Science and Technology (HUST), Luoyu Road 1037, Wuhan, 430074 People’s Republic of China; 2 College of Materials and Chemical Engineering, China Three Gorges University, Daxue Road 8, Yichang, 443002 People’s Republic of China; 3 Hubei Key Laboratory of Optical Information and Pattern Recognition, School of Optical Information and Energy Engineering, School of Mathematics and Physics, Wuhan Institute of Technology, Guanggu 1st Road 206, Wuhan, 430205 People’s Republic of China

**Keywords:** Mo_2_C, MoS_2_, Chemical vapor deposition, Asymmetric metal contacts, Photodetector

## Abstract

Self-powered devices are widely used in the detection and sensing fields. Asymmetric metal contacts provide an effective way to obtain self-powered devices. Finding two stable metallic electrode materials with large work function differences is the key to obtain highly efficient asymmetric metal contacts structures. However, common metal electrode materials have similar and high work functions, making it difficult to form an asymmetric contacts structure with a large work function difference. Herein, Mo_2_C crystals with low work function (3.8 eV) was obtained by chemical vapor deposition (CVD) method. The large work function difference between Mo_2_C and Au allowed us to synthesize an efficient Mo_2_C/MoS_2_/Au photodetector with asymmetric metal contact structure, which enables light detection without external electric power. We believe that this novel device provides a new direction for the design of miniature self-powered photodetectors. These results also highlight the great potential of ultrathin Mo_2_C prepared by CVD in heterojunction device applications.

**Electronic supplementary material:**

The online version of this article (10.1007/s40820-019-0262-4) contains supplementary material, which is available to authorized users.

## Highlights

- 2D Mo_2_C was produced by a modified chemical vapor deposition method, and 2D Mo_2_C-Au was formed as an asymmetric contact structure with a large work function difference.
- Mo_2_C/MoS_2_/Au photodetectors powered by asymmetric contact structure can work under self-powered condition with a responsivity of 10^−1^ mA W^−1^. The detection performance of the photodetectors can be stable for at least 110 days.

**Electronic supplementary material:**

The online version of this article (10.1007/s40820-019-0262-4) contains supplementary material, which is available to authorized users.

## Graphical abstract

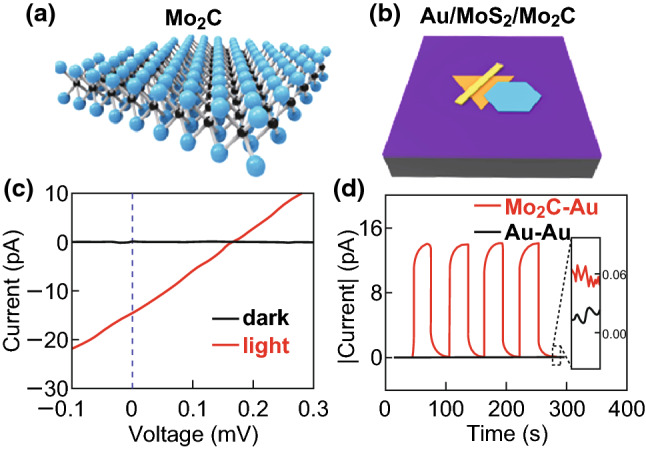

## Introduction

Self-powered and high-sensitivity miniature photodetectors are very attractive for a broad range of applications, spanning the imaging, environmental monitoring, and biomedical diagnostics fields [1–7]. Two-dimensional (2D) materials offer the opportunity to produce the performance of self-powered and high-sensitivity miniature photodetectors [8–14]. 2D material-based photodetectors with various structures have been widely reported [15–23]. The fabrication of asymmetric metal contact structures based on 2D materials is an effective method to obtain self-powered photodetectors [24, 25]. One advantage of self-powered devices driven by an asymmetric contact is that they are suitable for long operation times [26]. The large difference in work function between asymmetric source and drain contact metal is beneficial for the fabrication of efficient detection devices [27, 28]. However, common metal electrodes have a high work function, which makes it difficult to create an asymmetric structure with a large work function difference between the contacts. Obtaining ultrathin and stable electrode materials with a low work function is still a major challenge. Some ultrathin transition metal carbides (TMCs) with low work function may be potential candidates for the fabrication of asymmetric contact structures [29–32]. Nevertheless, the materials prepared by chemically etching methods (known as MXenes) always exhibit surface terminations, such as hydroxyl, oxygen, or fluorine, which affect their properties and structures [33]. The small lateral size and thermal instability of MXene also limits its application in planar optoelectronic devices [34]. The chemical vapor deposition (CVD) method offers a new way to synthesize large-area 2D TMCs (such as Mo_2_C, TaC, and WC) and their derivatives, without surface terminations [35–40]. Ultrathin Mo_2_C crystals with a work function of 3.8 eV, prepared by the CVD method, are both thermally (they remain stable in air at 200 °C) and environmentally stable under H_2_ plasma treatment [41–43]. It has been reported that CVD-grown ultrathin Mo_2_C (CVD-Mo_2_C) has significant superconducting properties, with a high critical temperature [35] and is suitable for the preparation of asymmetric metal structures. However, dense nucleation sites are often formed during the Mo_2_C preparation by the CVD method, which limits the lateral growth of Mo_2_C.

In this work, we first optimized the CVD process for preparing Mo_2_C. The use of graphene-intercalated copper can reduce the number of Mo_2_C nucleation sites in the initial growth stage, thereby facilitating the synthesis of large-area Mo_2_C. Then, a self-powered Mo_2_C/MoS_2_/Au device with an asymmetric metal contact structure was fabricated. The large work function difference (1.3 eV) between Mo_2_C and Au favors the fabrication of an efficient MoS_2_-based photodetector. The Mo_2_C/MoS_2_/Au-structured device could enable the detection of light without external electric power. We believe that this novel device provides a new direction for the design of self-powered miniature detectors. The present work also highlights the great potential of ultrathin CVD-Mo_2_C in electrode applications.

## Experimental and Computational Methods

### Preparation of MoS_2_

Monolayer triangular MoS_2_ was synthesized through an atmospheric pressure CVD system with a two-temperature-zone tube furnace. Thoroughly cleaned *n*-type SiO_2_ (300 nm) on Si was used as the substrate. Molybdenum trioxide powder (Mo source) and sulfur powder (S source) were vaporized at temperatures of 850 and 180 °C, respectively, for the synthesis of MoS_2_ on the SiO_2_ substrate. Finally, the furnace was cooled down to room temperature naturally.

### Preparation of Mo_2_C

Three-layer, 25-μm-thick Cu foil (Alfa Aesar, 99.95% purity) was cut into pieces and placed on a 50-μm-thick Mo foil (Alfa Aesar, 99.95% purity). The Cu/Mo substrate was placed in a single-temperature-zone CVD system and heated to above 1096 °C under 200 sccm Ar (type I method, see below). The other Mo foil was placed near the Cu/Mo substrate (type II method). CH_4_ (5 sccm) and H_2_ (300 sccm) were introduced into the chamber to grow the Mo_2_C crystals. Finally, the tube furnace was naturally cooled to room temperature before collecting the Mo_2_C sample.

### Device Fabrication

After cleaning the Mo_2_C on Cu/Mo with H_2_ plasma to remove graphene from the surface of the sample [43], the Mo_2_C on the Cu/Mo substrate was placed face down on the silicon oxide wafer on which MoS_2_ was grown. A 1 M (NH_4_)_2_S_2_O_8_ aqueous solution was then used for etching the Cu layer, following which the Mo_2_C on the substrate surface dropped onto the silicon oxide wafer. After the sample was dried, a photoresist was spin-coated on the Mo_2_C–MoS_2_ sample at 4000 rpm for 60 s. After drying at 90 °C for 1.5 min, the target site for the vapor deposition of a gold electrode on the Mo_2_C-MoS_2_ was exposed with a 200 mW cm^−2^ laser. Then, the sample was placed in the developing solution and allowed to stand for 1 min. After rinsing the developing solution with deionized water, gold was evaporated onto the sample. Finally, we obtained the multifunctional and horizontally structured Mo_2_C/MoS_2_/Au sample by washing away the remaining photoresist with acetone.

### Theoretical Calculations

Structural relaxations and electronic calculations were performed by first-principles simulations based on density functional theory, as implemented in the CASTEP package [44]. The exchange–correlation interaction was treated within the generalized gradient approximation (GGA), using the Perdew–Burke–Ernzerhof (PBE) functional and a plane-wave basis with a kinetic energy cutoff of 500 eV [45, 46]. The long-range van der Waals interactions were considered using the DFT-D2 dispersion correction proposed by Grimme [47]. The atomic positions and cell vectors were relaxed until the maximum force and maximum stress tolerance were less than 0.01 eV Å^−1^ and 0.02 GPa, respectively. Vacuum gaps of at least 15 Å were used to minimize the interactions between adjacent images of the single-layer structure. The reciprocal space was sampled with dense grids of 16 × 16 × 1 (for structural optimizations) or 20 × 20 × 1 (for accurate band structure calculations) k-points in the Brillouin zone. The lattice constant, Mo–S bond length, and S–Mo–S bond angle of MoS_2_ after the structural optimization were 3.218 Å, 2.437 Å, and 80.63°, respectively. The calculated bond length of 3.218 Å is slightly different from that of the optimized lattice structure reported by Saha et al. [48] (*a* = *b* ≈ 3.19 Å).

### Characterization

Raman spectroscopy (LabRAM HR800, He–Ne laser excitation at 532 nm) was used for structural characterization. Optical images were acquired by a Leica DM4000 M microscope. Field-emission scanning electron microscopy (FE-SEM, FEI Nova Nano-SEM 450) and transmission electron microscopy (TEM, Tecnai G220 U-TWIN) were used for investigating the morphology and structure of the samples. The current–time characteristics of the photodetector were measured by a low-temperature cryogenic probe station (Lake Shore CRX-6.5 K), a semiconductor parameter analyzer (Keithley 4200-SCS), and a light source (Energetiq EQ-1500).

## Results and Discussion

**Fig. 1:**
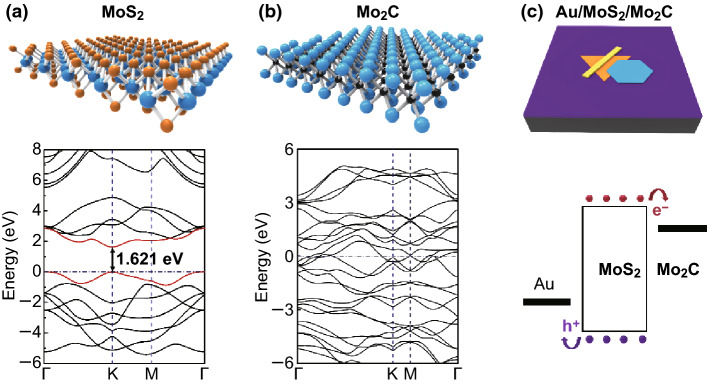
Schematic illustration (top) and calculated electronic band structure (bottom) of **a** monolayer MoS_2_ and **b** monolayer Mo_2_C. **c** Schematic diagram (top) and energy band diagram (bottom) of MoC_2_/MoS_2_/Au device

A schematic illustration and the calculated electronic band structure of monolayer MoS_2_ are shown in Fig. 1a. The calculated electronic band structure shows that the band gap of monolayer MoS_2_ is 1.62 eV, which can efficiently absorb light with a wavelength below 765 nm. Figure 1b shows a schematic illustration and the calculated electronic band structure of monolayer Mo_2_C. Mo_2_C exhibits good metallic properties, similar to other TMCs without surface terminations. A schematic diagram of the Mo_2_C/MoS_2_/Au device and its energy band structure is shown in Fig. 1c; the device exhibits an asymmetric metal contact structure. The monolayer MoS_2_ absorbs light and internally generates electron–hole pairs. The photogenerated electrons and holes move to molybdenum carbide and gold, respectively, under the potential difference induced by the asymmetric metal contacts.

**Fig. 2:**
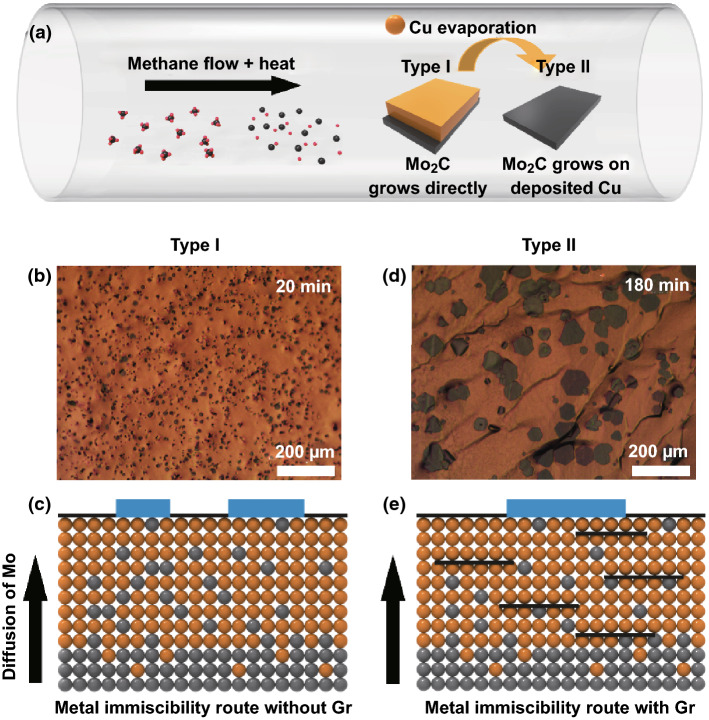
**a** Schematic diagram of the CVD method to grown Mo_2_C. In the type I method, copper foil is placed on molybdenum foil to directly grow molybdenum carbide. In the type II method, the molybdenum foil is placed near the copper foil. Copper evaporates at high temperatures and adsorbs on the molybdenum foil for the growth of Mo_2_C. **b** Optical image of Mo_2_C prepared by the type I method with a growth time of 20 min. **c** Schematic diagram of the type I Mo_2_C growth process. **d** Optical image of Mo_2_C prepared by the type II method. **e** Schematic diagram of the type II Mo_2_C growth process. The black line represents graphene

A schematic diagram of the CVD growth process of Mo_2_C is shown in Fig. 2a. Methane is rapidly cracked at 1096 °C, providing the carbon source for the growth of Mo_2_C. There are two ways to grow molybdenum carbide on a copper/molybdenum film substrate using the principle of metal immiscibility. In the first method (denoted as “type I”), the copper foil is placed on molybdenum foil as a growth substrate for Mo_2_C crystals. Mo atoms diffuse toward the surface of the copper foil at high temperatures and eventually react with carbon atoms to form molybdenum carbide. This method can produce highly crystalline 2D Mo_2_C on the in situ grown graphene [36]. However, under the growth conditions of the type I method, the rapid diffusion of Mo atoms in the copper foil at high temperature results in the formation of dense Mo_2_C nucleation sites on the surface of the copper foil. Figures 2b and S1 show optical images of Mo_2_C prepared by the type I method with growth times of 20 and 180 min, respectively. The optical images reveal the presence of high-density, small-area molybdenum carbide on the surface of the copper foil. As shown in Fig. 2c, in the type I growth mode, the diffusion process of Mo atoms from the molybdenum foil to the liquid copper surface is unhindered. We developed a new Mo_2_C growth method, denoted as “type II.” In this method, copper foil is not placed on top of the molybdenum foil, but next to it. The distance between the Mo and the Cu foil is 2 mm. Copper vapor is deposited on the molybdenum foil under high-temperature and methane gas flow conditions. Graphene is continuously formed on the liquid copper surface during the copper vapor deposition process. Finally, Mo_2_C is formed on the surface of the copper film. Figure 2d shows an optical image of Mo_2_C prepared by the type II method with a growth time of 180 min. The comparison of Fig. 2b and S1 shows that the molybdenum carbide crystals prepared by the type II method exhibit significantly fewer nucleation sites as well as larger areas. Figure S2a, b shows the thickness of the Cu layer in the Cu/Mo substrate after 180 min of Mo_2_C growth by the type I and type II methods, respectively. The Cu layer thickness in Figure S2a is thicker than that in Figure S2b. Generally, the thinner the copper layer in the substrate, the higher the amount of molybdenum carbide grown on its surface [49]. However, under the same growth conditions, the copper layer obtained by the type II method is thinner and less molybdenum carbide is formed. Therefore, we can infer that the growth process of molybdenum carbide in the type II method is similar to that shown in Fig. 2e, which shows the diffusion process of molybdenum atoms in copper. Graphene has a very fast growth rate. During the deposition of copper vapor onto the molybdenum foil, a large amount of graphene crystals is formed on the copper surface and further covered by the newly deposited copper, eventually forming a graphene-intercalated copper structure. The intercalation of graphene in copper hinders the diffusion of molybdenum atoms, resulting in a small number of Mo atoms diffusing into the copper surface. Graphene on the copper surface also has a passivation effect on the nucleation of molybdenum carbide. Finally, only a small number of Mo_2_C nucleation sites are formed on the copper surface, which leaves room for the formation of larger Mo_2_C crystals. Figure S3a shows an optical image of Mo_2_C with graphene, which transferred on the SiO_2_ from the Cu/Mo substrate. Figure S3b shows the Raman spectra of graphene corresponding to the point marked “a” in Figure S3a.

**Fig. 3:**
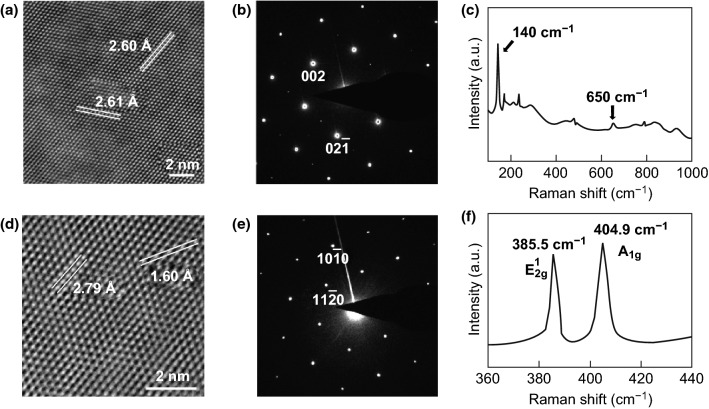
**a** High-resolution TEM image of Mo_2_C, with space group *Pbcn*. **b** SAED pattern along the [$\bar{1}00$] zone axis. **c** Raman spectrum of MoS_2_. **d** High-resolution TEM image of MoS_2_, with space group *P6*_*3*_/*mmc*. **e** SAED pattern along the [0001] zone axis. **f** Raman spectrum of MoS_2_

Figure 3a, b shows a high-resolution TEM image of Mo_2_C and the selected area electron diffraction (SAED) pattern along the [$\overline{1}$ 00] zone axis, respectively. The interplanar distances of the (002) and ($02\overline{1}$) planes are 2.60 and 2.61 Å, respectively. Figure 3c shows the Raman spectrum of Mo_2_C; the two characteristic Raman peaks of Mo_2_C crystals are located near 140 and 650 cm^−1^, respectively, in good agreement with previous reports [43, 49]. Figure 3d, e shows the high-resolution TEM image of MoS_2_ and the corresponding SAED pattern along the [0001] zone axis, respectively. The interplanar distances of the ($10\overline{1} 0$) and ($11\overline{2} 0$) planes are 2.79 and 1.60 Å, respectively. Figure S4 displays optical images of Mo_2_C and MoS_2_ on SiO_2_. The Raman spectrum of MoS_2_ is shown in Fig. 3f, where the $E_{{2{\text{g}}}}^{1}$ and $A_{{1{\text{g}}}}^{{}}$ peaks are found at 385.5 and 404.9 cm^−1^, respectively. The difference between the positions of the two peaks (19.4 cm^−1^) is characteristic of monolayer MoS_2_ [50]. To confirm the stoichiometry of Mo_2_C, the elemental distribution and electron energy-dispersive spectroscopy (EDS) data of Mo_2_C are shown in Figures S5 and S6, respectively.

**Fig. 4:**
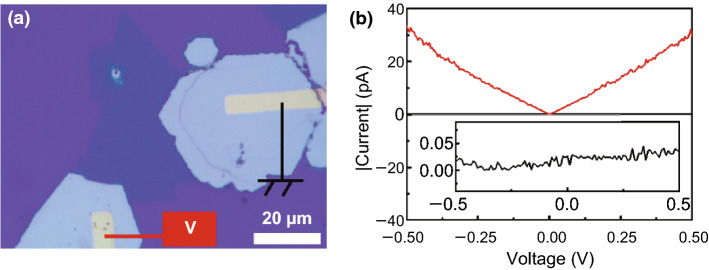
**a** Optical image of the Mo_2_C/MoS_2_/Mo_2_C device. **b** Dark (black) and light (red) *I*–*V* curves of the Mo_2_C/MoS_2_/Mo_2_C photodetector. The inset image shows the magnified dark *I*–*V* curve of the photodetector

In order to investigate the characteristics of Mo_2_C as an electrode material, we prepared a Mo_2_C/MoS_2_/Mo_2_C device and tested its *I*–*V* characteristics in the dark and under illumination. Figure 4a shows the optical image of the Mo_2_C/MoS_2_/Mo_2_C device. At zero bias, the device does not have photodetection capabilities. At small bias voltages, the dark current of the device does not increase significantly, while the light current shows a marked increase, as shown in Fig. 4b. This result indicates that Mo_2_C can form a good metal–semiconductor (MS) contact with MoS_2_, although the Mo_2_C/MoS_2_/Mo_2_C device with symmetrical electrode structure cannot provide self-powered detection of light.

**Fig. 5:**
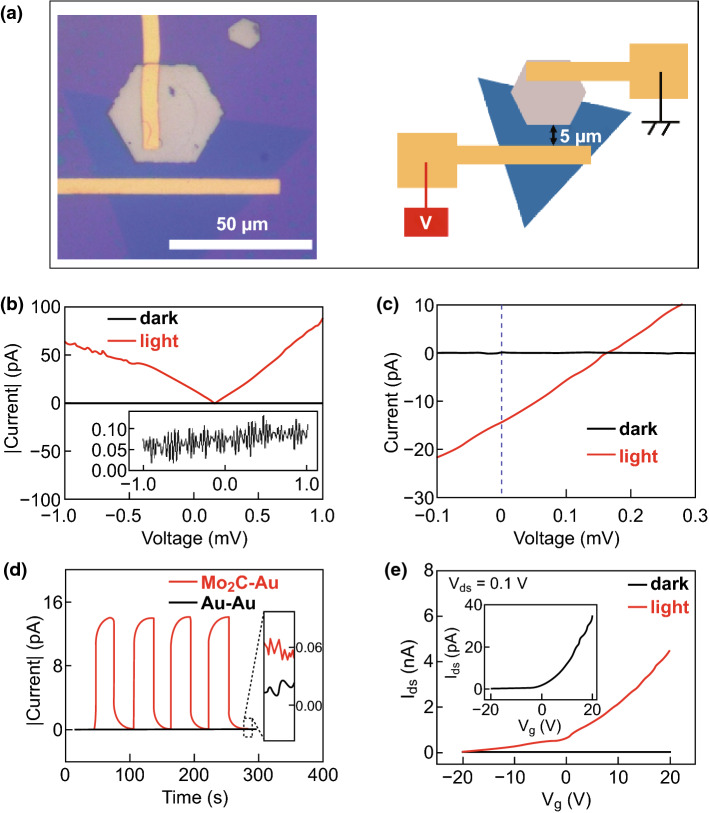
**a** Optical image and schematic illustration of the Mo_2_C/MoS_2_/Au device. **b** |*I*|–*V* curves of Mo_2_C/MoS_2_/Au devices with (red) and without (black) 80 mW cm^−2^ white light irradiation. The inset image shows the magnified dark current. **c** Magnified *I*-*V* curves of Mo_2_C/MoS_2_/Au devices with a bias voltage range of − 0.1 to 0.3 mV. **d** Photocurrent response of self-powered Mo_2_C/MoS_2_/Au (red) and Au/MoS_2_/Au (black) devices under 80 mW cm^−2^ white light irradiation. The image in the inset shows the magnified dark current. **e** Transfer characteristic curves of the photodetector with and without white light irradiation

Figure 5a shows the morphology and a schematic illustration of the Mo_2_C/MoS_2_/Au photodetector. The size of the channel between the gold electrode and the Mo_2_C material is approximately 5 μm. Au and Mo_2_C are connected to the source and drain electrodes, respectively. Figure 5b shows the |*I*|–*V* curves of Mo_2_C/MoS_2_/Au photodetectors. No significant increase in dark current is observed at small bias voltages. At bias voltages of 1 and − 1 V, different light currents are generated in the device as a result of the asymmetry of the work functions of the two electrodes. Figure 5c shows the magnified *I*–*V* curves of Mo_2_C/MoS_2_/Au photodetectors in the dark and under irradiation. Under 80 mW cm^−2^ illumination, the photodetector exhibits an open circuit voltage of 0.16 mV and a short-circuit current of approximately 15 pA. Figure 5d shows that the Mo_2_C/MoS_2_/Au device exhibits a clear photocurrent response under irradiation with 80 mW cm^−2^ white light. Since the electrodes at both ends have identical work functions, no significant photocurrent response is detected for the Au/MoS_2_/Au device. Figure 5e shows the transfer characteristics of the Mo_2_C/MoS_2_/Au devices on 300 nm SiO_2_ and 300 μm *n*-type Si substrates. The inset shows an enlarged view of the transfer curve in the dark. The transfer curves show that the devices exhibit *n*-type field effect transistor (FET) characteristics, due to the sulfur vacancies and the surface states of MoS_2_. Since electrons in n-type MoS_2_ are more likely to move from the valence to the conduction band under forward bias conditions, a larger source–drain current is generated when the device is illuminated under forward bias.

**Fig. 6:**
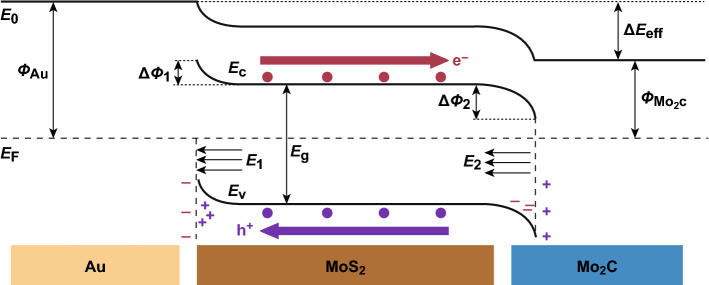
Schematic band energy diagram of the Mo_2_C/MoS_2_/Au device

Figure 6 shows the band energy diagram of Mo_2_C/MoS_2_/Au, without considering the surface states of MoS_2_. As MoS_2_ is connected to Au and Mo_2_C, a potential difference *∆E*_eff_ is generated between Au and Mo_2_C. The value of *∆E*_eff_ corresponds to the difference between the work functions of Au (*Φ*_Au_ = 5.1 eV) and Mo_2_C (*Φ*_Mo2C_ = 3.8 eV): *∆E*_eff_ = (5.1–3.8) eV = 1.3 eV. The contact between *n*-type MoS_2_ and Au forms an electron blocking layer at the interface. Because MoS_2_ induces the accumulation of a large amount of holes in the electron blocking layer and of electrons on the Au surface, its energy band is bent upward at the interface and generates a built-in electric field (*E*_1_) directed from MoS_2_ to Au. The work function of MoS_2_ on SiO_2_ is 4.49 eV [51]. In the contact between MoS_2_ and Au, the barrier height on the molybdenum sulfide side (*∆Φ*_1_) caused by the band bending is 0.6 eV (corresponding to the difference between the work functions of MoS_2_ and Au). The contact of MoS_2_ with Mo_2_C leads to the formation of an electron anti-blocking layer at the interface. Electrons and holes accumulate on the MoS_2_ and Mo_2_C side, respectively, and generate a built-in electric field (*E*_2_) from Mo_2_C to MoS_2_. The barrier height (*∆Φ*_2_) produced by the MoS_2_–Mo_2_C contact of is − 0.7 eV. Due to this band structure, electrons photogenerated in MoS_2_ can smoothly move from MoS_2_ to Mo_2_C instead of Au. The transfer direction of photogenerated holes in molybdenum sulfide is opposite to that of photogenerated electrons.

**Fig. 7:**
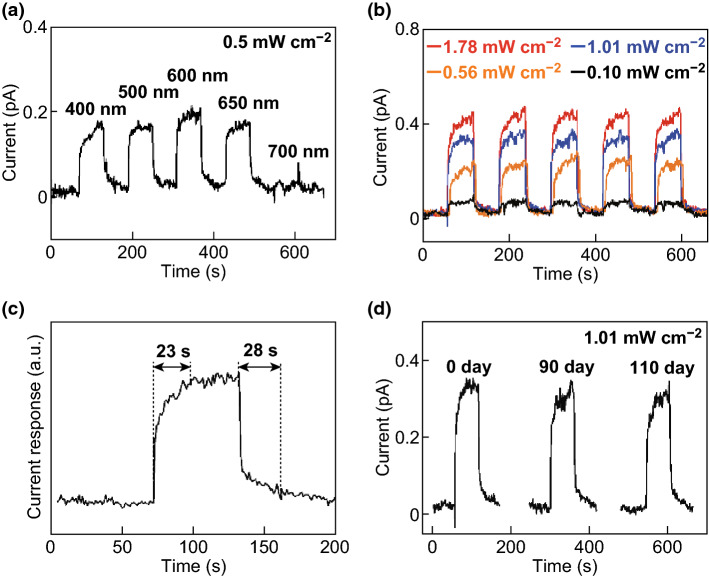
**a** Photodetector responses to light with various wavelengths and energy density of 0.5 mW cm^−2^. **b** Photodetector responses to 600 nm light of different intensities. **c** Response and recovery times of the photodetectors. The intensity of the incident light (600 nm) is 0.56 mW cm^−2^. **d** Long-term performance of the Mo_2_C/MoS_2_/Au photodetector under illumination with 600 nm light

Figure 7a shows that the response of the device to 0.5 mW cm^−2^ light at 600 nm is slightly higher than that at 400, 500, and 650 nm. The Mo_2_C/MoS_2_/Au photodetector exhibits almost no response to 700 nm light. Figure S7 shows the current responses of the Mo_2_C/MoS_2_/Au photodetector to light of various wavelengths. When the wavelength of the incident light is increased to 700 nm, the photocurrent response of the device drops rapidly; therefore, the band gap of MoS_2_ is about 1.77 eV. Figure 7b shows the photodetector response to 600 nm light of different intensities. The photocurrent increases with increasing light intensity. At an energy density of 1.78 mW cm^−2^ and a wavelength of 600 nm, the device has a switching ratio of approximately 10 and a responsivity of approximately 10^−1^ mA W^−1^. As shown in Fig. 7c, the response and recovery times of the photodetectors are 23 and 28 s, respectively. Moreover, Fig. 7d shows that the responsivity of the photodetector remains approximately constant over 110 days, indicating the high reliability and stability of self-powered Mo_2_C/MoS_2_/Au photodetectors.

The characteristics of the Mo_2_C/MoS_2_/Au device are compared to those of photodetectors with different structures in Table 1. Au/MoS_2_/Mo_2_C shows a larger difference between the work functions of the electrodes than that of other asymmetric structures. The self-powered Au/CVD-MoS_2_/Mo_2_C device has a slightly faster response speed than Au/CVD-MoS_2_/Au which should work at bias voltage. Although the responsiveness of the Au/MoS_2_/Mo_2_C devices measured in this study is not outstanding, the responsivity of photodetectors driven by an asymmetric contact can be improved by reducing the spacing between the electrodes or decorating light-absorbing materials with quantum dots [27, 52]. We thus believe that the Mo_2_C/semiconductor/Au configuration is an effective asymmetrical structure for self-powered photodetectors.

**Table 1:** Comparison of the characteristics and performance of Mo_2_C/MoS_2_/Au photodetectors with photodetectors with other structures

Device	Work function difference	Photoresponsivity	Rise/decay time	References
Au/graphene/Al	0.82 eV	4.9 mA W^−1^	< 7 ns/< 7 ns	[24]
Au/graphene/Ti	0.77 eV	52 mA W^−1^	–	[27]
Au/CVD-MoS_2_/Au	5 V (bias voltage)	2.97 × 10^4^ A W^−1^	30 s/32 s	[52]
Au/exfoliated-MoS_2_/Au	1 V (bias voltage)	0.42 mA W^−1^	50 ms/50 ms	[53]
Au/MoS_2_/Mo_2_C	1.3 eV	0.1 mA W^−1^	23 s/28 s	This work

## Conclusions

In conclusion, our results show that the use of graphene-intercalated copper to prepare Mo_2_C can affect the diffusion of Mo atoms in copper and reduce the number of Mo_2_C nucleation sites in the initial growth stage, thereby facilitating the synthesis of large-area Mo_2_C. The physical and chemically stable Mo_2_C has a low work function and is well suited for the preparation of asymmetric metal contact structures. The MoS_2_-based photodetectors powered by asymmetric contact structure with large work function difference can detect light of wavelength below 700 nm without external power. The responsivity of Mo_2_C/MoS_2_/Au photodetectors is approximately 10^−1^ mA W^−1^ under light irradiation at 600 nm and 1.78 mW cm^−2^. The response and recover times are 23 and 28 s, respectively. This novel device may open new avenues for the design of self-powered multifunctional miniature devices. The present study also reveals the great potential of ultrathin CVD-Mo_2_C in electrode applications.

## Electronic supplementary material

Below is the link to the electronic supplementary material.

Supplementary material 1 (PDF 444 kb)
