# Supplementary material for: MoS2-Based Photodetectors Powered by Asymmetric Contact Structure with Large Work Function Difference
Source: Nanomicro Lett. 2019 Apr 16;11:34. doi: 10.1007/s40820-019-0262-4 (PMC7770726; doi:10.1007/s40820-019-0262-4)
Supplement: Supplementary file 1 — Supplementary material 1 (PDF 444 kb) [file 40820_2019_262_MOESM1_ESM.pdf]

Supporting Information for

MoS_2-Based Photodetectors Powered by Asymmetric Contact Structure with Large Work Function Difference

Zhe Kang[1], Yongfa Cheng[1], Zhi Zheng[1], Feng Cheng[1], Ziyu Chen[1], Luying Li[1], Xinyu Tan[2,*], Lun Xiong[3], Tianyou Zhai[1], Yihua Gao[1,3,*]

[1]Center for Nanoscale Characterization & Devices (CNCD), Wuhan National Laboratory for Optoelectronics (WNLO) & School of Physics & School of Materials Science and Engineering, Huazhong University of Science and Technology (HUST), LuoyuRoad 1037, Wuhan 430074, People's Republic of China

[2]College of Materials and Chemical Engineering, China Three Gorges University, Daxue Road 8, Yichang 443002, People's Republic of China

[3]Hubei Key Laboratory of Optical Information and Pattern Recognition, School of Optical Information and Energy Engineering, School of Mathematics and Physics, Wuhan Institute of Technology, Guanggu 1st Road 206, Wuhan 430205, People's Republic of China

*Corresponding authors. E-mail: gaoyihua@hust.edu.cn (Yihua Gao), tanxin@ctgu.edu.cn (Xinyu Tan)

Supplementary Figures

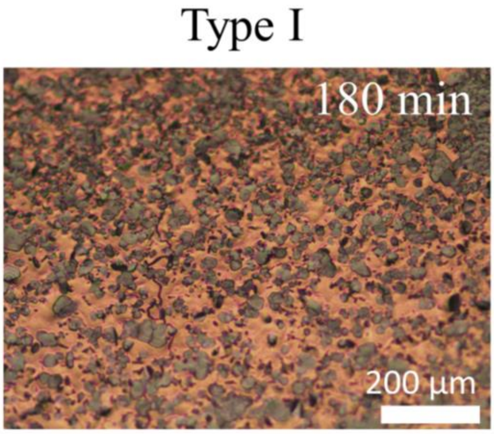

Fig. S1 The optical image of Mo_2C prepared by Type I method with the growth time of 180 min

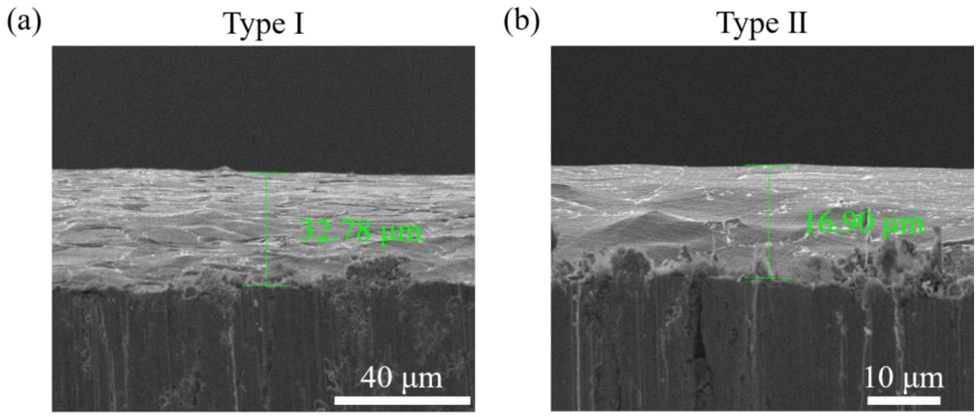

Fig. S2 Thickness of Cu in the Cu/Mo substrate after 180 min of growth of Mo_2C by **a** Type I and **b** Type II method

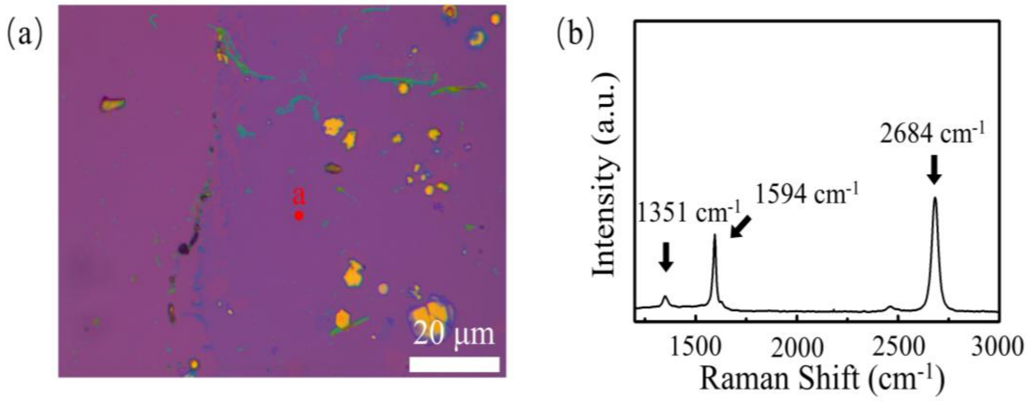

Fig. S3 **a** The optical image of Mo_2C with Graphene on SiO_2 substrate. **b** The Raman spectra of graphene obtained by testing the point a of **a**

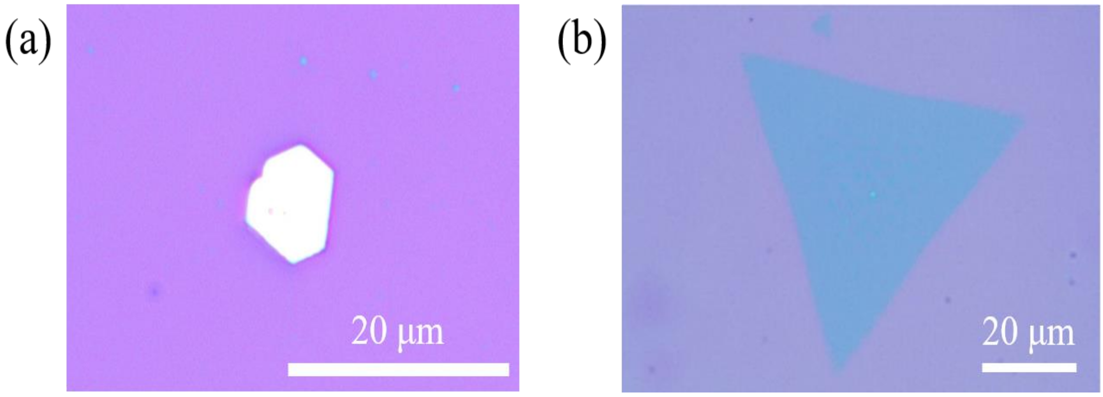

Fig. S4 The optical image of **a** Mo_2C on SiO_2. **b** MoS_2 on SiO_2

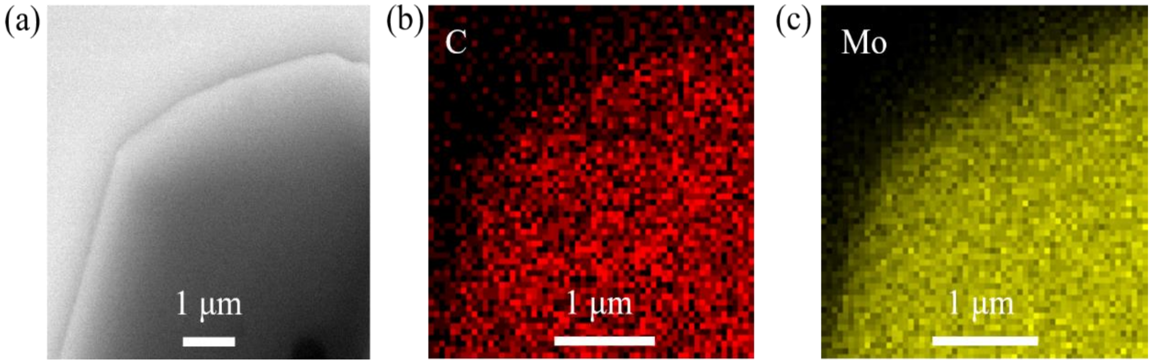

Fig. S5 The elemental distribution of Mo_2C. **a** STEM image, **b** C distribution, and **c** Mo distribution of Mo_2C

Element	Weight %	Atomic %	Uncert. %	Correction	k-Factor
C(K)	5.99	33.74	0.91	0.28	3.685
Mo(K)	94.00	66.25	2.62	0.98	4.047

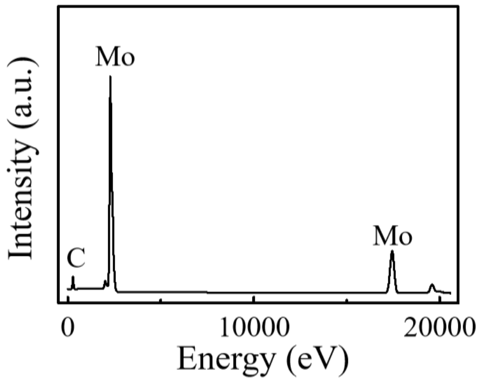

Fig. S6 The EDS of Mo_2C

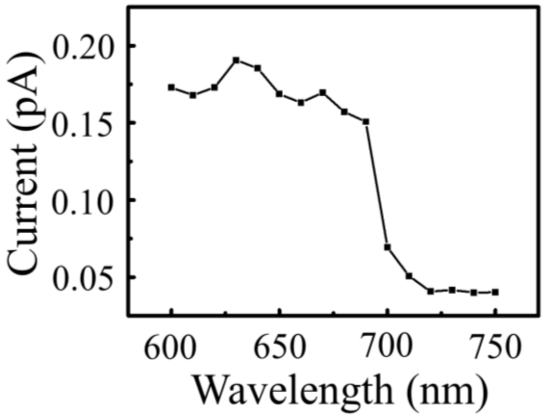

Fig. S7 The current responses of $Mo_2C/MoS_2/Au$ photodetector to light with various wavelength
